# Robot Art, in the Eye of the Beholder?: Personalized Metaphors Facilitate Communication of Emotions and Creativity

**DOI:** 10.3389/frobt.2021.668986

**Published:** 2021-07-15

**Authors:** Martin Cooney

**Affiliations:** Center for Applied Intelligent Systems Research (CAISR), Department of Intelligent Systems and Digital Design, Halmstad University, Halmstad, Sweden

**Keywords:** socially assistive robotics, robot art, affective robotics, robot-assisted therapy, human-robot interaction, social robotics, artificial emotions, artificial creativity

## Abstract

Socially assistive robots are being designed to support people’s well-being in contexts such as art therapy where human therapists are scarce, by making art together with people in an appropriate way. A challenge is that various complex and idiosyncratic concepts relating to art, like emotions and creativity, are not yet well understood. Guided by the principles of speculative design, the current article describes the use of a collaborative prototyping approach involving artists and engineers to explore this design space, especially in regard to general and personalized art-making strategies. This led to identifying a goal: to generate representational or abstract art that connects emotionally with people’s art and shows creativity. For this, an approach involving personalized “visual metaphors” was proposed, which balances the degree to which a robot’s art is influenced by interacting persons. The results of a small user study via a survey provided further insight into people’s perceptions: the general design was perceived as intended and appealed; as well, personalization via representational symbols appeared to lead to easier and clearer communication of emotions than via abstract symbols. In closing, the article describes a simplified demo, and discusses future challenges. Thus, the contribution of the current work lies in suggesting how a robot can seek to interact with people in an emotional and creative way through personalized art; thereby, the aim is to stimulate ideation in this promising area and facilitate acceptance of such robots in everyday human environments.

## 1 Introduction

The current article proposes that social robots will follow the path of smartphones in becoming prevalent, once they too appear to provide various forms of value at reasonably low cost (hereafter referred to as the “smart phone hypothesis”). This will involve helping not just with everyday tasks and emergencies, but also with fulfilling our social needs, to help us to flourish (e.g., (Fitter and Kuchenbecker, 2018; Block and Kuchenbecker, 2019; Nakagawa et al., 2020)). In particular, social needs for affection and self-fulfillment strongly involve emotions and creativity (Maslow, 1943), which have been described as “final frontiers” in artificial intelligence (Picard, 1995; Colton and Wiggins, 2012). (Some might argue that it would be impossible for robots to engage in such interactions, as emotions and creativity are human traits; here, an alternative perspective is adopted, that these terms refer to observable “processes,” rather than traits that someone or something might or might not possess. Moreover, considering the growing number of artificially intelligent systems targeting applications that were previously thought to be restricted to humans–from complex games like “Go” to writing, composing, and depicting–it seems possible that this line of research could one day affect how we think about ourselves [e.g., if emotions and creativity can no longer be used as a differentia specifica for humans, what might be next for Plato’s “featherless biped” (Hodges et al., 2010)?).) Here we focus on one such emotional and creative activity that people of all ages and cultures can enjoy, art-making; painting together with others can positively affect a person’s restfulness, self-image, stress tolerance, and vital signs–facilitated by processes of self-exploration, self-fulfillment, catharsis, and self-categorization (Stuckey and Nobel, 2010). From a therapeutic perspective, such interactions with robots could also help to alleviate the rising shortage of human caregivers and growing problem of persisting loneliness, which has been linked to high costs and ruinous health outcomes, and is being exacerbated by isolation caused by COVID-19.

**FIGURE 1:**
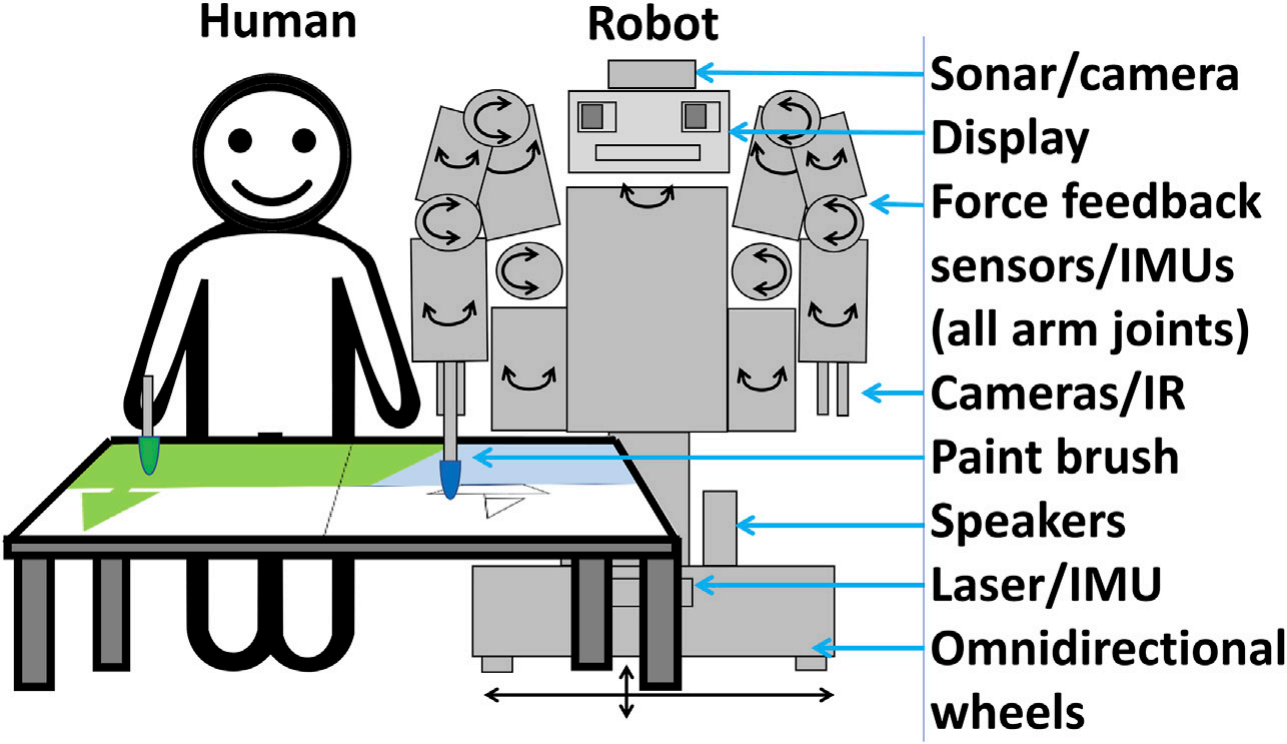
Basic concept. A social robot could interact with people in emotional and creative contexts such as art-making, that provide enjoyment or therapeutic value, given some strategies for personalization and expression through art.

To get started in this complex and challenging scenario, a basic outline of the design space was required: A primary concern was to identify norms and underlying “codes” that could provide value for various users–but personalization was also deemed to be important, as somaesthetic experiences like art-making are highly idiosyncratic (Kerruish, 2017). Furthermore, art can take various forms, such as representational or abstract, which could be perceived differently. Therefore, the goal of the current article was to gain insight into the basic “lay of the land” for how to design a socially assistive painting robot, including such perspectives on personalization and art form; the basic concept is illustrated in Figure 1, and some definitions are also provided in Table 1.

**TABLE 1:** Some definitions of terms used in this article.

Socially assistive robot	An embedded computing system, comprising sensors and actuators which afford some semi-autonomous, intelligent, or human-like qualities, intended to interact to support people’s well-being
Well-being	A subjectively perceived state, related to happiness, life satisfaction, and quality of life, encompassing physical, psychological, and social factors (hedonic and eudaimonic), and linked with positive emotions and creativity
Emotion	A complex psycho-physical process involving cognitive appraisals, subjective feelings, somatic symptoms, and affect displays, related to sentiment and mood. (Emotion is typically encoded in a simplified manner via dimensions or categories in computers (“the affective gap”); one important interactive form of emotion is empathy, the capability to demonstrate recognition of and caring for another’s emotions, which relates to “emotional contingency” or emotional relatedness.)
Creativity	A way of operating characterized by novelty, not something one has or doesn’t have (Gershgorn, 2016)
Personalization	A process of adapting to a target, also referred to as customization or tailoring, which has been observed to have positive effects on engagement and trust (Sillence et al., 2006), especially in areas where people are highly different
Symbol	Some representation of a concept, person, or thing. (Here the term does not refer to symbolic art, which was a reaction against realism.)
Abstract	Nonrepresentational, in the sense that people and objects cannot be clearly discerned–rather the art uses shapes and colors to evoke impressions

To address the goal, a speculative prototyping approach was adopted, combining the insights of both engineers and artists, to derive a theoretical design and practical implementation, which could be checked and refined via a small user study: Speculative design facilitates the formation of concrete ideas and problem detection in expansive, ambiguous design spaces (Dunne and Raby, 2013). An exploration solely by artists might have trouble in building the robotic recognition and behavioral capabilities required for interaction, whereas engineers might lack crucial insight into how to communicate effectively through art. Likewise, purely theoretical studies can miss identifying real world challenges, and practical implementations require grounding in ideation to be relevant; for this, “mid-fidelity prototyping” was used to balance speed of investigation with accuracy of insights. Thus, the aim was not to build a finished product or to reveal detailed behavioral mechanisms through rigorous experimentation. Rather, the contribution of the current work lies in exploring this highly complex scenario involving emotions, creativity, robots, and art, and reporting the questions and challenges that arise, toward stimulating discussion and informing next steps in this promising area, as part of an ongoing, larger effort.

The remainder of this article is organized as follows: Section 1.1 discusses some related work, identifying gaps related to general art-making strategies and personalization approaches; in particular, two basic categories of robots are identified, based on how much their art is influenced by the behavior of interacting persons. Section 2.1 describes an interaction design derived from our collaborations with artists that strives to balance perceived emotions and creativity, from both theoretical and practical perspectives. How people perceive the design was explored in a small user study with a survey, described in Section 2.2. Then, various quantitative and qualitative results are presented in Section 3 and discussed in Section 4, along with ideas for next steps.

### 1.1 Related Work

The idea of robots that can make art has long fascinated (Herath et al., 2016), and interest in robot art has been growing recently, also as a means to explore new ways of thinking about interactive robots (St-Onge, 2019). For this, collaborations between artists and engineers have been observed to have positive synergistic effects (St-Onge et al., 2011). Some ongoing work has even involved the first testing of an art-making robot at a hospital, resulting in some positive initial feedback (Herath et al., 2020). The design of such interactions could be enriched through insight into general robot art-making strategies and personalization approaches.

#### 1.1.1 General Strategies

Many art-making robots have been created by artists and engineers, for various audiences to see or interact with; here only a few examples can be described. Robot morphologies are diverse, comprising robot arms, as well as humanoid, vehicular, flying, and even animal-like robots (e.g., “Picassnake” (Seo and Young, 2017)). One commonality is that typically some kind of “seed” is selected to guide the robot’s art; whereas humans continuously gather rich, multimodal information through complex interactions over long periods, that can be used to inspire art, current robots tend to make art based on more limited data, rulesets, and capabilities. As well, two common kinds of art-making strategy can be described as interactively “exogenous” or “endogenous”: Exogenous robots are tools that are dependent on humans to control them, such that some physical human signal like motion or sound results in some physical motion by the robot, allowing human creativity to be directly leveraged. By contrast, endogenous robots mostly operate independently of a human artist, in a stand-alone fashion, and creativity is drawn from some other source such as random numbers and events.

A typical example of an exogenous system could be a photocopier, printer, or plotter, which require and are directly controlled by a person’s input. More uncommonly, prototypes can be controlled via facial images, eye movements, body movements, or sound: For example, a person can get their portrait done by showing their face to a humanoid robot (Calinon et al., 2005) or arm robot like “Obsessive Drafter,” which draws on a large wall (Obsessive Drafter, 2021). Eye movements can be used to control a painting robot built by Faisal and colleagues (e.g., blinking to change colors) (Smith and Sayers, 2015). A person can dance to get Tate Corso’s “Manibus” to summarize their motions as a painting (Crowder, 2017). Also, a person can play music to get WUSTL’s Action Jackson to paint like Pollock (Action Jackson, 2007).

In contrast, an extreme example of an endogenous system that does not react to people could be a robot arm that is programmed to paint one specific scene, and nothing else. More complex systems can use aleatoric uncertainty to generate interesting art (Creative Machine, 2014): For example, Brown’s “Computer Assisted Drawing” used random numbers with plotters in the mid-1970s, toward realizing autopoietic “art that makes itself” (Brown, 2003). Tinguely’s Meta-Matics machines scrabbled with a pen over paper (Metamatics, 2014). Graffitizer used the randomness of ink drips on a wall to introduce complexity in its art, stemming from minute vibrations and variance in the amount of ink on a brush (Graffitizer, 2013). Additionally, Moon’s drawing robot traced random map images from Google Maps, as well as the trajectory of a cricket’s motions in a box (Moon, 2013). What was unclear from the literature was how these strategies compare and which would be desirable within the current context, in which a physically collocated robot and human co-create art.

#### 1.1.2 Personalization

The idiosyncratic nature in which art is perceived also suggests the importance of personalization. Some related knowledge has been elucidated in the field of empirical aesthetics in regard to beauty evaluations (“beauty evaluation” refers to judging the degree of attractiveness of an artwork (Mallon et al., 2014)). For example, studies have reported on how general and personal taste influence human perception depending on the degree to which symbols are abstracted (Leder et al., 2016). But, how a robot can generate art which appears to express certain emotions such as happiness or sadness to an individual is less clear. For example, Van Gogh’s Starry Night might seem dreamy or despairing; Bosch’s triptych fascinating or frightening; and Dali’s depiction of melting memory resigned or regretful, in the vein of Shelley’s Ozymandias. Correctly identifying the meaning of a person’s art could improve rapport, whereas mistakes could damage the trust a person has in a robot, especially in a therapy setting. More generally, personalization offers improved services which are easier to use, more satisfying, and more persuasive; such effects have also been seen in practice, for example, in positive emotions resulting from personalization of a robot in a language class (Gordon et al., 2016).

Personalization is conducted by applying knowledge of users to a system’s behavior, and can be seen inter alia on the web in recommendations, advertisements, search results, and social media content; in interactive learning systems, through question selection based on “knowledge tracing”; in product development in the form of data-driven “personas”; and in commercial products such as mugs, shirts, books, and statues that use photos, names, or 3D data. Typically, personalization in the digital realm involves user models and profiles: a model describes how a user can be represented in terms of some properties of interest like name or gender, which is used to structure a user profile, the data for a specific user or group. Data can be obtained explicitly in a “user-driven” manner by directly querying users, or implicitly in a “system-driven” manner, where either approach has benefits and demerits; explicit user-driven approaches can overwhelm users, who also might not know exactly what they want from the start, whereas system-driven approaches can incorporate restrictive hidden biases.

Some concerns in defining a user model include what properties to consider and how to structure them, as well as how the data will be obtained and used. Many possible properties could affect the perception of emotions in visual art, with commensurately many possible model configurations. A naïve or brute force method might seek to obtain data for every single concept that could be expressed through art from a person, which is not likely to be practical. At a higher level of abstraction, stereotypes can be used; for example, properties felt to be important for the emotional rating of art that were included in the OASIS affective image dataset include age, gender, geographic location, race, ideological self-placement, and socioeconomic background (highest level of education, and household income) (Kurdi et al., 2017). For communication on social media, Zhao et al. proposed considering also social context, a person’s previous emotional state, and influence of location (e. g., a photo taken in an entertainment venue might be happy, whereas a photo taken at a funeral might be sad), as well as personality via the Big Five model (Zhao et al., 2016, Zhao et al., 2019). Similarly, Rudovic et al. proposed a model to encode how autistic children show emotions in their audiovisual and physiological behavior, at three levels–culture, gender, and individual (Rudovic et al., 2018)–although other configurations might exist: for example, could the first layer be gender rather than culture, and what happens when other variables like age are considered?

Thus, the literature did not clarify how to design a socially assistive painting robot, which uses a personalized model to visually convey emotions; it seems like few studies have investigated the degree of overlap in people’s perceptions of emotions in art, or how this can be modeled (also in terms of different forms of art, like abstract or representational) and how this can be transparently integrated into a system that can move from theoretical concepts to a concrete painting.

## 2 Methods and Materials

### 2.1 Painting Together With a Human

The current section summarizes some of our previous work, which involved first identifying a basic scenario informed by the related literature (Cooney and Menezes, 2018). For simplicity, the basic scenario selected for exploration was dyadic (one human and robot), visual (non-verbal), and conducted over a single session (e.g., 10 min), with free choice of what to paint. Requirements for a robot included the capability to move safety near people and make art (human-like reach and cameras), as well as a familiar interface which could support social interactions (such as a “skeuomorphic” humanoid form). (A skeuomorph is an artifact that retains some ornamental features from some original form from which it was derived; e.g., to make it easy for a user to infer how it can be used (Skeuomorph, 2020). The humanoid form in an interactive social robot is here referred to as “skeuomorphic” because, unlike the humans after which they are designed, such robots typically do not need to be human-shaped, but rather use this form to leverage people’s familiarity in interacting with humans, as a way to enable communication. In other words, humans can guess that a robot can see and speak if it has eyes and a mouth, etc). Based on these requirements, a Baxter robot was chosen. The human and Baxter robot can paint side-by-side, or face-to-face if the canvas is not vertical, but lying on a desk or table; for our study, the latter formation was preferable due to the robot’s width–which can range from approximately 0.8–2.6 m depending on whether arms are tucked in or fully extended to its sides–and improved reach and visibility.

Based on this scenario, our group of artists and engineers followed a mid-fidelity research prototyping approach to gain some insight (Cooney and Berck, 2019). Thus, the artists guided the exploration of various different scenarios and setups, both interactively exogenous and endogenous, while the engineering students controlled the robot. Both artists contributed with their knowledge of art, in advising how the robot could seek to paint, which included materials and strategies, providing examples of sketches and paintings intended to express various emotions, and participating in various meetings, art-making sessions, demo events, and data collection (e.g., for a Brain-Machine Interface). Some additional information can be seen in Appendix A.

One idea that emerged from the sessions was that an exogenous robot could paint in a contingent way to indicate empathy. Contingent in Human-Robot Interaction (HRI) means “related” or “connected,” such that there appears to be “a correspondence of one’s behaviour to another’s behaviour” (Pitsch et al., 2009). The simplest way to suggest that a robot can relate its art to what a human has painted is direct mimicking, or painting the same thing the human has painted. This felt interesting, in that the robot seemed to be perceptive and to attribute importance to the human’s art. But, merely copying also felt like the robot was a mere machine, not a partner.

Another enjoyable interaction involved an artist painting together with the robot on a shared canvas, where the robot painted endogenously, independently of the artist. This felt creative and artistically interesting, since the artist could improvise, draw ideas, and build on what the robot painted. However, the lack of awareness from the robot of the human’s behavior, and thereby the lack of a connection or bond, also felt like a human working with a machine, not a partner. Thus, both strategies felt limiting due to their one-way nature.

This led us to consider if a robot could produce art that balances exogenous and endogenous components to appear both empathetic and creative; thus, a robot could base its art partially on what a human does, and partially on its own intentions (e.g., to help the human to feel good). In general, such a need exists, for robots to interact effectively in relation to social and emotional aspects, to improve their competence and support positive user experiences (Lowe, 2019). For example, trust is desirable for a robot to be effective. Similarly, an ability to both consider other’s behavior and produce something new could be useful in indicating some degree of intelligence–as robots that are perceived as intentional agents with a mind (i.e., “mind perception”) are more likely to be treated as a partner and be the recipients of empathy, morality or prosocial behaviors (Wiese et al., 2017). Furthermore, creatively reshaping a human’s emotional expression could lead to more stimulating, thought-provoking, and meaningful art–like in the “responsive art” approach, in which an art therapist provides visual feedback based on a person’s art, toward exploring its meaning and achieving positive experiences. Also, the usefulness of appearing to balance exogenous and endogenous components in a robot’s behavior to indicate agency has been previously suggested in HRI, within a related context of how a robot can direct attention toward interacting people (Kroos and Herath, 2012).

As a first step to explore such a strategy, the use of a “visual metaphor” was proposed: namely, that a robot can paint something that is similar in emotional meaning but different in creative expression. This is loosely in line with the concept of the “adjacent possible” (Tria et al., 2018) that describes first-order combinations of existing ideas, and based on our idea that different symbols can be painted to express the same emotion. For example, if a person paints a quiet forest, the robot could paint something else relaxing, like a babbling brook. Grieving people could be painted beside a remorseful grave scene. A snake poised to strike could be painted beside a threatening gun. Alternatively, bright balloons could be painted beside a festive stack of presents. This idea is general and can also be applied to abstract art. For example, if a person uses diagonal lines to express arousal, a robot could instead use a warm color to achieve a similar effect. Thus, the term “metaphor” here is used to describe such a different symbol that is intended to express a similar emotional meaning. For example, a painted circle could be seen as an abstract representation of a balloon, but might be useful to paint, not because there is any specific meaning in depicting a balloon, but rather as a metaphor to express an emotion of joy which leverages generally shared perceptions of the meanings of symbols.

This concept thus has both an emotional and a creative side, and art in general can be seen as comprising both sides, as discussed in “Expression Theory” (Khatchadourian, 1965). From the emotional perspective, our artists noted that art is created for various reasons; at any given time, an artist might be interested in expressing their own emotions, or in eliciting particular responses from an observer. For the context of a robot engaging in therapy or entertainment, the latter is the focus.

**FIGURE 2:**
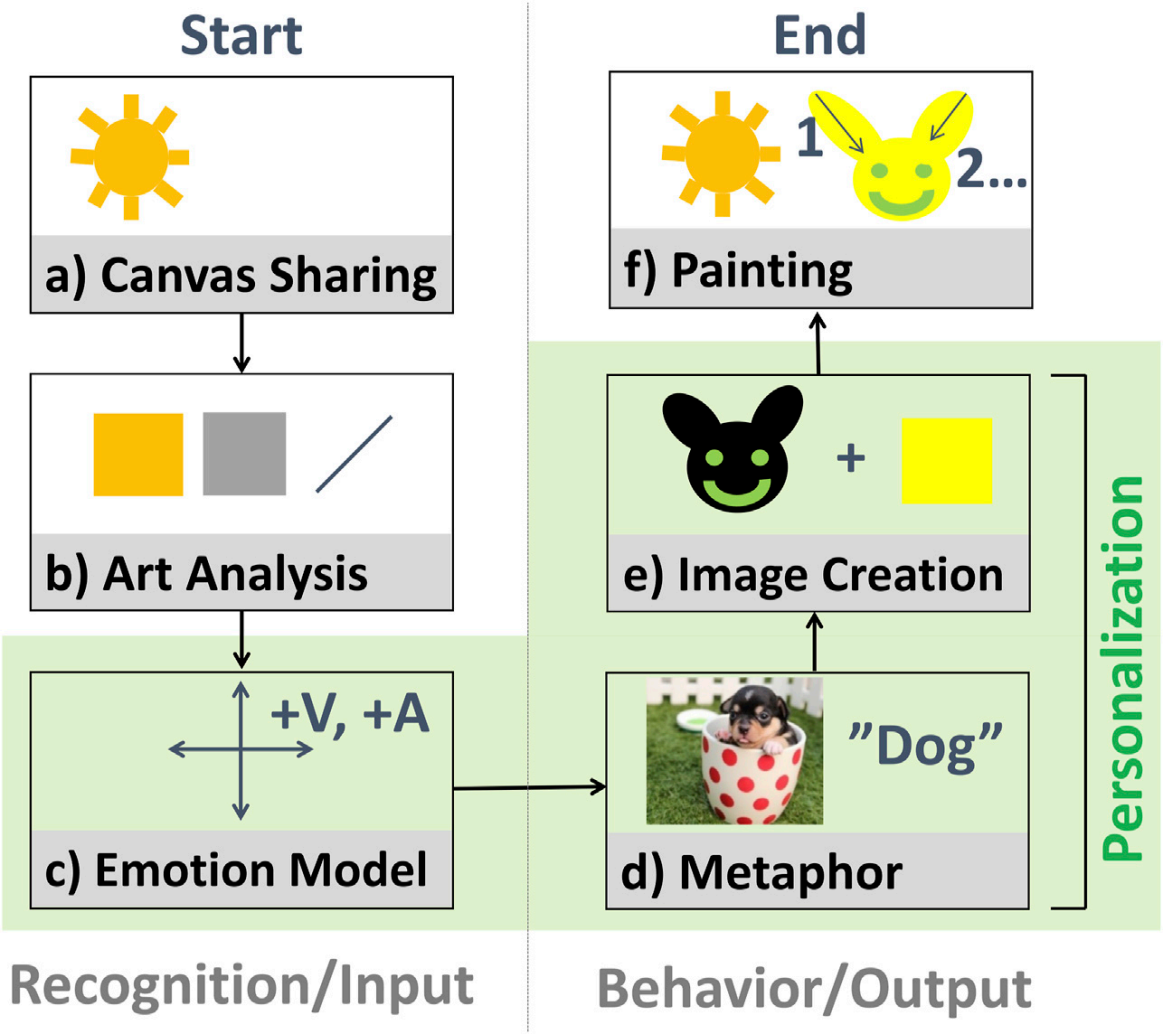
A process flow for using visual metaphors to convey emotional contingency (empathy) and creativity in art that a robot creates with a human.

How then to incorporate this concept into a semi-autonomous interaction? As shown in Figure 2, a robot can detect when and where to paint; infer the emotional meaning of a person’s art by analyzing colors, lines, and composition; select a contingent metaphor with an affective image database; generate an image based on the metaphor; and paint the image:

• (a) Canvas sharing. To avoid interrupting a person while they paint, a robot can either predict a person’s intentions and motions and plan accordingly, or more simply, follow a turn-based interaction design. In the latter case, a person could indicate that it is the robot’s turn to paint haptically, verbally, or visually–each with some potential demerits. For example, it might be difficult for a person with a cognitive disorder to haptically control a robot, which is not required in typical interactions with humans. Controlling a robot verbally using CMU PocketSphinx (Pocketsphinx, 2021) or Google Speech (Google Speech, 2021) might require ability to enunciate clearly at adequate volume; in our exploration we also noted that a non-trivial strategy would be required for the robot to deal with its own sounds, from speech to noise from actuators, as well as environmental noise. Moreover, visual control, via foreground detection through OpenCV (OpenCV, 2021)–either static or adaptive–could be used to detect a person’s hands or brush moving over the canvas; but, during our simplified exploration, challenges were observed with illumination (flickering, shadows, and occlusions) and slight movements of the robot’s arm with the camera, which generated false positives. Another alternative could be to combine various modalities for robustness. For this simplified initial exploration, a turn-based design with haptic control was implemented, in which a person presses a button on the robot’s arm to indicate when it is the robot’s turn to paint.
• (b) Art and Biosignal Analysis. To seem contingent, the robot should perceive what the human has expressed. Given that paintings are primarily visual, a camera, either on the robot or in the room, can be used to analyze a person’s painting. Algorithms will likely become increasingly capable of high-level summarization and observation; currently, low-level and mid-level analysis is typical, which involves detecting colors, lines/shapes, composition, and depicted objects. One complementary alternative is to also detect biosignals linked to emotion, such as heart or respiratory rate, skin conductance, muscle current or brain activity; we used the latter in our previous work, which required the user to wear a Brain-Machine Interface. For our current prototype, a camera on the robot was used. Conversion in OpenCV to HSV (Hue-Saturation-Value) space was conducted to color-pick six basic hues and calculate their average intensity, while using the Hough Transform to detect lines.
• (c) Emotion Inference. Next, the robot should seek to infer the emotional meaning underlying the physiological signals detected, such as visual expression or brainwaves. A generic model based on some emotion model could be used, or a personalized profile with information on how a person associates art with emotions, if available. How such a profile could be constructed based on querying a user is considered in the next section.

For our initial prototype, some simplified heuristics related to the visual arts were used in conjunction with a generic dimensional model of emotion: A linear combination of features based on detected colors and lines was used to calculate valence and arousal, for which Ståhl’s model was used to calculate a contribution of each hue by area: this model provides a way to link colors to emotions, by rearranging Itten’s color circle to fit Russell’s Circumplex Model of Affect (Ståhl et al., 2005). Intensity also influenced valence, with light being positive and dark being negative, and the incidence of diagonal lines affected arousal.

• (d) Metaphor Selection. The next step involved finding a new way to express a detected emotional meaning. Nouns with a similar emotion connotation could be looked up via a large sentiment lexicon such as Affective Norms for English Words (ANEW) (Bradley and Lang, 1999), SentiWordNet (Baccianella et al., 2010), or WordNet-Affect (Strapparava and Valitutti, 2004), possibly with a concreteness rating to ensure that the noun can be expressed visually in a recognizable way; or, more simply, an affective image database could be used. A challenge identified was polysemy, which also related to bias and variance in data: for example, not all images of dogs will induce the same emotional responses, given disparities in canine size and aggressiveness, as well as human preferences and beliefs. A personalized profile could also be used here.

For our prototype, a simplified capability was implemented to search two affective image databases, namely the Open Affective Standardized Image Set (OASIS (Kurdi et al., 2017), which has 900 open-access color photographs assigned with normative emotion ratings, and the International Affective Picture System (IAPS) (Lang et al., 2008), which has 1,195 rated color photographs. For example, looking for happy, relaxed, sad, and angry emotions resulted in images showing dogs, flowers, gray yarn, and injuries in OASIS, and skydiving, nature, a cemetery, and mutilation in IAPS, respectively.

• (e) Image Generation (Reification). A plan is required to move from a metaphor like “dog” to a specific image of a dog to paint. For example, a generative approach, in conjunction with examples of dog images from Google Image Search or ImageNet (Deng et al., 2009), could be used to generate a “unique” image of a dog. This image could then be abstracted, and the color and lines modified to more clearly convey an emotion, or to less resemble previous images that the robot has drawn. A personalized profile could also be used for this step.

For initial exploration, Deep Convolutional Generative Adversarial Networks (DC-GANs) were used to develop compositions. Some challenges related to time were encountered, that prohibited the kind of online interaction we wished to achieve: web-scraping many images required much time; even with small MNIST-sized (LeCun et al., 1998) grayscale images, the algorithm took hours to generate new images; and the algorithm did not function automatically (a human was needed to select appropriate images from the output). Two simpler alternatives include applying filters to some automatically-combined web-scraped images, or asking artists to come up with images for some typical metaphors and manipulating these to create new images by reflecting, rotating, swapping colors, etc.

• (f) Painting Plan and Execution. Next, the robot should move to paint the target image on the canvas. This part of the process, although also challenging, has seen much focus in previous work, e.g., using visual control loops (Lindemeier et al., 2015) (It is also possible to simplify complicated images before depicting them, like in the work of Wang and colleagues, who used a CycleGAN and genetic algorithm for image-to-image translation (Wang et al., 2019).) An advanced algorithm might seek to also factor time into account and only conduct a subset of the most important strokes before giving the human another turn to avoid long waiting times. For our prototype, our artists and students were asked to generate some motions for the robot to perform based on created images. (Additionally, it was explored how the prototype could also seek to show emotions through a face in its display, as well as motion curvature and velocity, and voice, during painting.)

Above, steps (C)-(E) allow for personalization. As a useful starting point for exploration, the current article proposes a Folksonomic-style model, in which open questions are used to capture symbols that exert a large emotional effect on an individual. A Folksonomy is a taxonomy formed by allowing users to add their own idiosyncratic tags to describe content (McLean et al., 2007); such a sparse and flat model can be useful in complex situations like the current one where it might be unclear how to construct a complete taxonomy, or what kind of architecture would be appropriate. Moreover, given the high variance in how people could choose to emotionally interpret “tricky” concepts, and the estimated difficulty in accurately estimating what someone is thinking without direct input, an approach based on user-driven self-disclosure was adopted. To avoid overwhelming users, the number of questions was restricted and, for simplicity, single emotions were considered. Mapping from emotions to paintings via personalized symbols was explored by combining results from lookups in an affective image database, followed by post-processing. In doing so, the concept for the personalization module involved accepting a user identity and target emotional state as input, and outputting a paintable image expected to elicit this emotional state in the identified user.

This overall process for art-making can be repeated over multiple turns, thereby closing the “affective loop”: perceiving emotions, acting, checking the result, and refining. A video describing this initial prototype is available online (Cooney, 2019).

### 2.2 User Study

In developing an initial prototype, the current article posited that people would rather interact with a robot that balances exogenous and endogenous content to appear both contingent and creative, rather than just one or the other, but arguments can also be made to the contrary: Since robot art systems today are often controlled by humans (exogenous) or paint independently without humans (endogenous), people might prefer a system that is not creative but is just controlled by them due to familiarity with using tools, as familiarity supports usability and technological acceptance, and control supports enjoyment; or conversely, people might prefer a system that is not contingent but generates good art regardless of human performance, and can surprise, kindle imagination, stimulate, and inspire. Moreover, questions existed about how people would perceive personalized art in various forms, such as abstract or representational.

To provide exploratory insight into these questions, a user study was conducted. As noted previously, the core approach in this article is speculative design, where often there is no user study (or even implementation); the user study here merely aimed to explore the above concepts from another perspective. For this, a simplified scenario was adopted in regard to robot strategies, the degree of personalization, the kind of art, and target emotions: For the robot’s art strategy, three systems were considered:

• System 1: Exogenous (dependent on the person’s art)
• System 2: Endogenous (independent of the person’s art)
• System 3: Proposed (balancing both dependent and independent elements)

Personalization was examined in a binary manner, as either generic or personalized, where the generic art was taken into account as a starting point when developing the personalized art. Likewise, the form of art was examined in a binary manner, as either abstract or representational. Since the aim was to obtain insight into how visual expressions can be personalized and the focus was not on the implementation, paintings were represented using sketches which require less time to produce, following the spirit of our prototyping approach. For target emotions, four representative discrete emotions were selected, one per quadrant in valence-arousal space, in line with Russell’s circumplex model (i.e., the corners of the “Affect Grid”): happy (high valence, high arousal), relaxed (high valence, low arousal), sad (low valence, low arousal), angry (low valence, high arousal) (Russell, 1980). (Three of these (happy, sad, angry) are also included in the six basic pan-cultural “Ekman” emotions, which do not include any relaxed emotion with high valence, low arousal (Ekman et al., 1969); a relaxed emotion was also represented for balance and due to its estimated importance in therapeutic applications.)

Various options exist for how to conduct a user study. As noted, the current article does not aim to verify or validate some complete solution in a formal lab experiment, which at the start would have been impractical and introduced many confounds (e.g., our prototype described in the previous section took several hours to generate images and was limited to dogs). Rather what was desired was a way to explore important concepts and gain initial insight to refine the design, like a survey. A survey offers a practical, safe way to acquire information that would be difficult to obtain by observation in order to form generalizations, especially given restrictions due to the COVID-19 pandemic; demerits include unsuitability when an understanding of historical context is required, bias due to non-respondents (less educated people are less likely to respond), intentional misreporting, and difficulty that respondents can experience in assessing their own perceptions (Glasow, 2005). Here these demerits were not prohibitive since historical context is not required, the intended sample population at our university is educated, intentional misreporting was not expected given that the topic is not controversial, and no better way appeared to exist to obtain feedback on respondents perceptions.

Thus, as a first step in our ongoing work, a simplified user study was conducted using an online survey: The main part of the survey checked our assumptions about a generic strategy and acquired data to build a personalized emotional profile; additionally, some extra insight was obtained into how participants perceived art generated using a Wizard of Oz approach based on their profile obtained in the first part of the survey.

#### 2.2.1 Participants

21 adults at our university (6 female, 15 male; average age: 34.0, SD = 9.5, from 10 countries, where Swedish nationality was most common, followed by Iranian and Indian) participated in the first and main step of the survey. This included both faculty members and students in two computer science master programmes, such that all participants had at least an undergraduate degree in engineering or science. No members of our team, and no artists or art students, took part; and, participants received no compensation.

#### 2.2.2 Ethics Statement

In Sweden, according to the ethics review act of 2003, additional formal approval by an ethics council is required for “interventions using methods intended to physically or mentally influence” participants, (SFS no 2003:460) (European Network of Research Ethics Committees, 2003). Since the goal of the survey was to observe and not to change participants' impressions in any way, and since sensitive personal information like race, politics, sexual behavior, or genetic/biometrics were not considered, the general principles described in the General Data Protection Regulation (GDPR, 2018) and Declaration of Helsinki (World Medical Association, 2018) were followed: The purpose of the study and basic approach were explained, and informed consent was obtained in writing, before beginning the survey. Gender, age, and nationality were temporarily collected to be able to report overall statistics that might influence perception of art; and in general precautions were taken to protect privacy and confidentiality, such as not storing names. Participation was completely voluntary and inclusive; the study design did not indicate the existence of an underrepresented group that should be targeted, but rather a range of different groups including students and faculty were invited to participate, and no vulnerable groups like minors under the age of 18 participated.

#### 2.2.3 Procedure

Participants were sent links to a Google Forms survey (Cooney, 2020b), which took approximately 20 min to complete.

**FIGURE 3:**
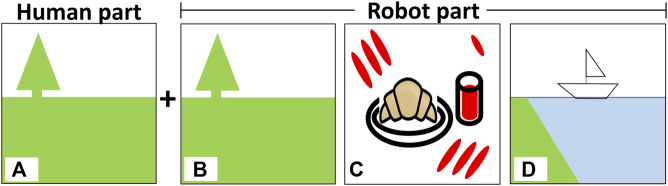
Images used to assess how people feel about a robot’s art-making strategy: **(A)** the human’s part, **(B)** the exogenous system 1 (the robot’s art is influenced entirely by what the human does), **(C)** the endogenous system 2 (the robot’s art is not at all influenced by what the human does), and **(D)** the proposed system 3 (the robot’s art seeks to express contingency and creativity by maintaining a balance of exogenous and endogenous concerns.

In the survey, participants answered questions about the robot’s strategy for making art, then disclosed information about what symbols, representational or abstract, elicited emotions for them. First, for the robot’s strategy, the participants were asked to inspect three images. For each image, the participants imagined that a human and robot has made art together, with the human’s art depicted on the left side and the robot’s art on the right side of the image. Each image represented an interaction with one of the three different versions of the robot–exogenous, endogenous, and proposed–as shown in Figure 3; for simplicity, the exogenous image was completely dependent on and similar to the person’s art, the endogenous image was completely independent of and different from the person’s art, and the proposed image contained a balance of both dependent and independent components. For each image, participants used a 5-point Likert scale to rate three statements:

• Q1 Contingency: “The robot’s art fits emotionally with the human’s art (i.e., the emotions expressed in both seem similar).”
• Q2 Creativity: “The robot’s art is creative.”
• Q3 Desire to use: “I would like to do art with this robot.”

Thus, the technical term “contingent” was rephrased to be more easily understood by participants. In the second half of the survey, participants described which representational symbols and abstract colors and shapes made them feel happy, relaxed, sad, or angry. The participants were also given a chance to add free comments, which were then coded.

## 3 Results

### 3.1 Art-Making Strategy

**FIGURE 4:**
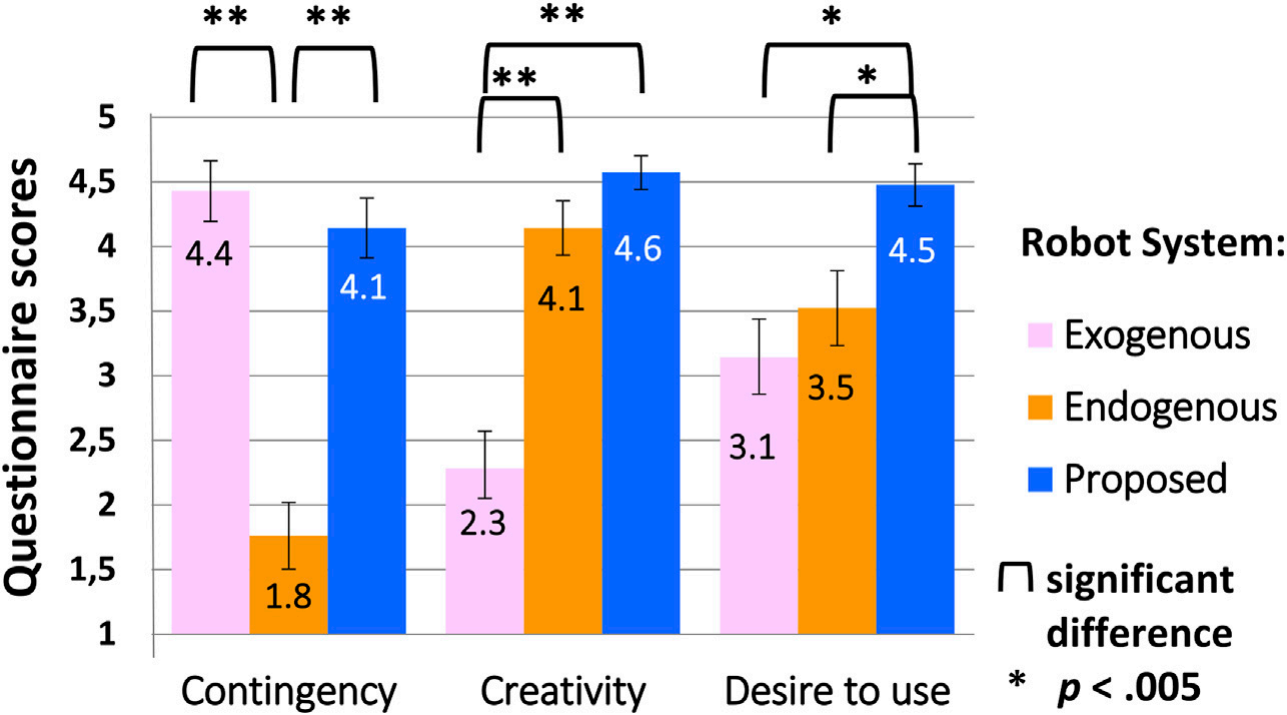
Questionnaire results for robot art-making strategy.

Figure 4 shows questionnaire results for how participants perceived the three art-making strategies.

The results were analyzed statistically. First, normality was assessed–which is a common assumption in some statistical tests–using Mardia’s coefficients for skewness and kurtosis (Kurtosis, 2015). The assumption was violated for skewness in question 1, at p<.001, which was also confirmed by visually checking the histogram. Therefore, non-parametric tests were used: in particular, Friedman tests, which are appropriate for ordinal data from Likert scales (McCrum-Gardner, 2008). These tests merely indicate if significant differences exist; to find where the differences exist, Wilcoxon signed-rank tests are typically used along with an adjustment for multiple comparisons such as Bonferroni adjustments, which simply divide the overall significance level by the number of hypotheses. Additionally, Yates’s chi-squared tests were applied to compare small amounts of categorical data, which are also common.

For question 1 about contingency, median ratings were 5, 1, and 4 for systems 1, 2, and 3, the exogenous, endogenous, and proposed systems; a Friedman test indicated that the systems were perceived differently in terms of contingency, $\chi^{2}\left(2\right)=32.771,p<.001$. Post-hoc analysis with a Wilcoxon signed-rank test and Bonferroni adjustment indicated that the endogenous system 2 was perceived to be less contingent than either of the other systems (compared to system 1: Z=−3.863,p<.001; compared to system 3: Z=−3.951,p<.001). No significant difference was observed between the exogenous and proposed systems (Z=−.907,p=.4).

Likewise, the systems were perceived differently in terms of creativity for question 2, with respective median values of 2, 4, and 5; $\chi^{2}\left(2\right)=26.800,p<.001$. A Wilcoxon signed-rank test with Bonferroni adjustment indicated that the proposed and endogenous systems were considered to be more creative than the exogenous system (Z=−3.337,p=.001; and Z=−3.659,p<.001). No difference was observed between the proposed and endogenous systems (Z=−1.897,p=.06), although with more data a trend might emerge, since there is variation in interpretations of what is creative (e.g., based on the degree to which usefulness is considered) (Diedrich et al., 2015).

Desire to make art with the robot also differed based on the robot’s strategy, with median scores 3, 4, and 5 for question 3; $\chi^{2}\left(2\right)=12.847,p=.002$. A Wilcoxon signed-rank test with Bonferroni adjustment indicated that participants would prefer to make art with the proposed version of the robot that appeared to be both contingent and creative, rather than just contingent or just creative (comparing system 3 and 1: Z=−3.135,p=.002; comparing system 3 and 2: Z=−2.797,p=.005). No difference was observed between the exogenous and endogenous systems 1 and 2 (Z=−0.917,p=.4). Furthermore, 14 out of the 21 participants said they would prefer to make art with the proposed system, compared to one with system 1, and five with system 2 (one person said they would prefer either system 2 or 3); a Chi Squared test with Yates correction indicated a significant difference in this stated preference, $\chi^{2}\left(2,N=20\right)=11.213,p=.004$. The one participant who preferred the dependent system stated that the reason was because it was “doing work as like as human” (sic). The participants who chose the independent system stated that it seemed “interesting,” “totally different,” and “unconventional,” arousing their intellectual curiosity (e.g., one participant wondered what pattern might underly its response and how reactive it was to a human’s behavior).

Thus, the basic premise of this work was supported: that a contingent and creative system combining exogenous and endogenous components might be more desirable as an art-making partner than a system which is only exogenous and contingent, or only endogenous and creative.

### 3.2 Emotional Triggers

Participants' self-disclosures about which symbols elicit emotions are collected in Table 2 and Table 3.

**TABLE 2:** Representational symbols disclosed by more than one participant as eliciting emotions. The top row indicates “typical” symbols described by more than one participant. Here the numbers beside each coded label indicate the number of mentioning participants, symbols that elicited more than one kind of emotion are indicated in bold, and comments in parentheses are given for clarification. The bottom row holds symbols indicated by only a sole participant.

Happy	Relaxed	Sad	Angry
Sports 9, family 8, food and drink 8, nature 8, traveling 5, sound (music) 4, work 3, visual leisure activities 3	Food and drink 10, visual leisure activities 8, sound (music) 8, nature 7, sports (exercise) 6, family 6, work (finishing work) 4, washing 3, rest 2	Failure 11, abusiveness 10, global problems (hunger, poverty, sickness) 8, family (missing) 7, injustice 5, nature (bad weather) 2, laziness 2	Abusiveness 9, injustice 9, ignorance 5, failure 3, sound (noise/shouting) 2, traffic 2
Freedom, gifts, bright colors, peace, truth, jokes, the smell of new books, happy endings, winning	Smiles, candles, silence, being in control	Bad news, seeing an “unhappy” plant, losing much money, witnessing others’ sadness	Pretentious people, communists, blood, crowds, inaction of those who can act, irresponsibility, pain, losing something, when someone special does not obey, being late, some trump supporters

**TABLE 3:** Abstract art elements disclosed by participants as eliciting emotions.

	Happy	Relaxed	Sad	Angry
Yellow	8	1	1	—
Orange	5	—	1	4
Pink	4	2	—	2
Purple	5	2	1	—
Green	7	8	—	1
White	6	8	2	2
Blue	8	7	1	—
Black	2	1	9	5
Red	2	—	3	10
Brown	—	—	8	—
Gray	1	1	1	1
Warm colors	1	1	—	1
Dark colors	—	1	1	1
(Subtotal)	48	31	27	26
Circle	8	8	3	1
Triangle	3	1	3	8
Square	2	2	5	2
Horizontal lines	2	9	1	1
Vertical lines	3	4	4	2
Diagonal lines	7	1	5	3
Curved	—	—	—	1
Everything	1	—	—	—
(Subtotal)	26	25	21	18
Total	74	56	48	44

As expected, there was a high degree of variation in participants' responses: 433 labels were provided (207 representational and 226 abstract labels, or approximately half-half). The labels were grouped into 69 categories, whereof 21 were abstract (8 shape and 13 color), and 48 were representational. Only three abstract categories were mentioned by only one person each, compared to 28 representational labels mentioned only by one person each, such that 20 representational categories contained the vast bulk of these labels, or 179 labels. Thus, on average each participant provided 20.6 labels, whereof 9.9 were representational and 10.8 were abstract (for forms and colors), constituting a response rate of 2.5 representational and 1.3 abstract labels per question. (Note, this count indicates the number of unique participants who mentioned a category as eliciting an emotion in a specific question; participants sometimes used synonyms, so one participant writing that “injust and unfair” circumstances elicit sadness would be counted just once toward the category “injustice” for this question.)

For representational symbols, the most frequent symbols were sports, family, food and drink, nature for eliciting happiness; food and drink, visual leisure activities, and music for relaxation; failure and abusiveness for sadness; and abusiveness and injustice for anger. Thus, there was overlap: 10 of 20 symbol categories were reported by more than one participant for only one of the emotion categories, whereas the other 10 were reported for more than one emotion. Such overlap usually occurred between positive or negative emotions, rather than between aroused or relaxed emotions: For example, food and drink could be both happy and relaxing. Nature and family were considered to cause either happy, relaxed, or sad emotions; poor weather and missing family was associated with sadness. Failures in some cases could be related to work (e.g., references to “programming errors”), but in general there were not enough details to link these two categories.

For abstract symbols, out of 13 color categories, twelve (92%) were mentioned as eliciting both positive and negative emotions, and all 13 were considered to be both calm and aroused. The only exception was brown, which was only negative and calm (sad). White, black, and gray were explicitly described as eliciting every emotion, although if only colors mentioned by at least two participants are considered, this drops to only white. Also, some participants mentioned “warm colors” instead of specifying individual colors, but if this is taken into account, then red, yellow, and orange also encoded for all four emotions. Nine categories were furthermore described as eliciting three out of four emotions.

The amount of overlap within individual participants' responses was also checked. Ten (48%) of the participants' responded in an overlapping way, in that one color could encode for more than one emotion (e.g., black for both sadness and anger), whereas the remaining eleven participants (52%) mentioned distinct colors for each emotion. In one extreme example, one participant wrote the same answer for all emotions: “graphite gray, black, white.”

15 participants (71%) indicated more than one color for one or more emotions, whereof two participants listed more than one color for each emotion, and one participant listed eight colors that felt happy. In contrast, six participants listed at most a single color that made them feel each emotion, with two participants mentioning a total of three cases in which no colors could express an emotion.

For shapes, there was likewise much variation and overlap. All categories mentioned by more than one participant (excluding “curved” and “everything”) were felt to elicit every emotion. Happiness was mostly shown by circles and diagonal lines, relaxation by horizontal lines and circles, sadness by squares and diagonal lines, and anger by triangles and diagonal lines. From the perspective of how each shape was perceived, circles seemed to be happy and relaxed, triangles to be angry, horizontal lines to be relaxed, diagonal lines were everything except relaxed (strongly showing various emotions), and vertical lines and squares seemed to be fairly uniformly spread out among the four categories.

Seven participants (33%) reported that a shape category expressed more than one emotion. (Two had overlap in saying that no shapes expressed certain kinds of emotions for them, like no shapes were negative.) Also, another seven participants (33%) mentioned two or more shapes for at least one emotion. Furthermore, four participants mentioned a total of eight cases in which no shapes could express an emotion.

Along the way, it was also observed that there were more responses for positive than negative emotions, with some participants mentioning that all shapes seemed positive, possibly due to their engineering background, or giving no responses at all regarding negative emotions. Additionally, various references were made to personal information that was not provided through the survey: e.g., “my cat,” “my programming errors,” “my home,” “my brother,” “my child,” “my mom,” and “my parents.” Without knowing more, it is difficult to depict such symbols visually: for example, should “my cat” be depicted as a giant Norwegian Forest cat, or as a tiny Munchkin?

### 3.3 Extra Insight: Assessment of the Sketches

The user study provided some general insight into how interactive art-making might be perceived, but left a question: Could personalized art based on self-disclosure more clearly convey emotions than using a general model? A concomitant challenge was that it was not clear if users would be able to accurately report which kinds of visually depicted concepts will best express various emotions to them. To gain some additional insight, four participants who completed the survey were asked to complete a follow-up survey (3 female, 1 male; average age: 30.2, SD = 5.0). These participants were again sent links to a Google Forms survey, which was this time personalized. In total, the follow-up survey took approximately 10 min to complete.

To prepare the follow-up survey, the participants' responses in the first survey were used to generate art in the form of eight personalized images (four abstract and four representational, thus two images per emotion). Personalized sketches were generated offline using the Wizard of Oz technique. The participants' responses were input into Google Image Search for images labelled free to reuse. To minimize the risk of using specific images that might communicate unintended signals, “clip art” was added to the search. For search queries where no appropriate image was found, synonyms were used. Images were manually selected to download and assembled into a single composition, before an art program effect was used as the last step to make the composition appear like a painting (Paint.NET) (Paint.NET, 2021).

The eight generic images were created once and used for all participants. Representational images were generated based on OASIS (Kurdi et al., 2017). The most extreme four OASIS images were identified corresponding to each emotion target. Abstract images were generated by our artists in line with our previous work based on heuristics including Ståhl’s model (Ståhl et al., 2005). Again, art program effects were used to ensure that the sketches looked like paintings.

**FIGURE 5:**
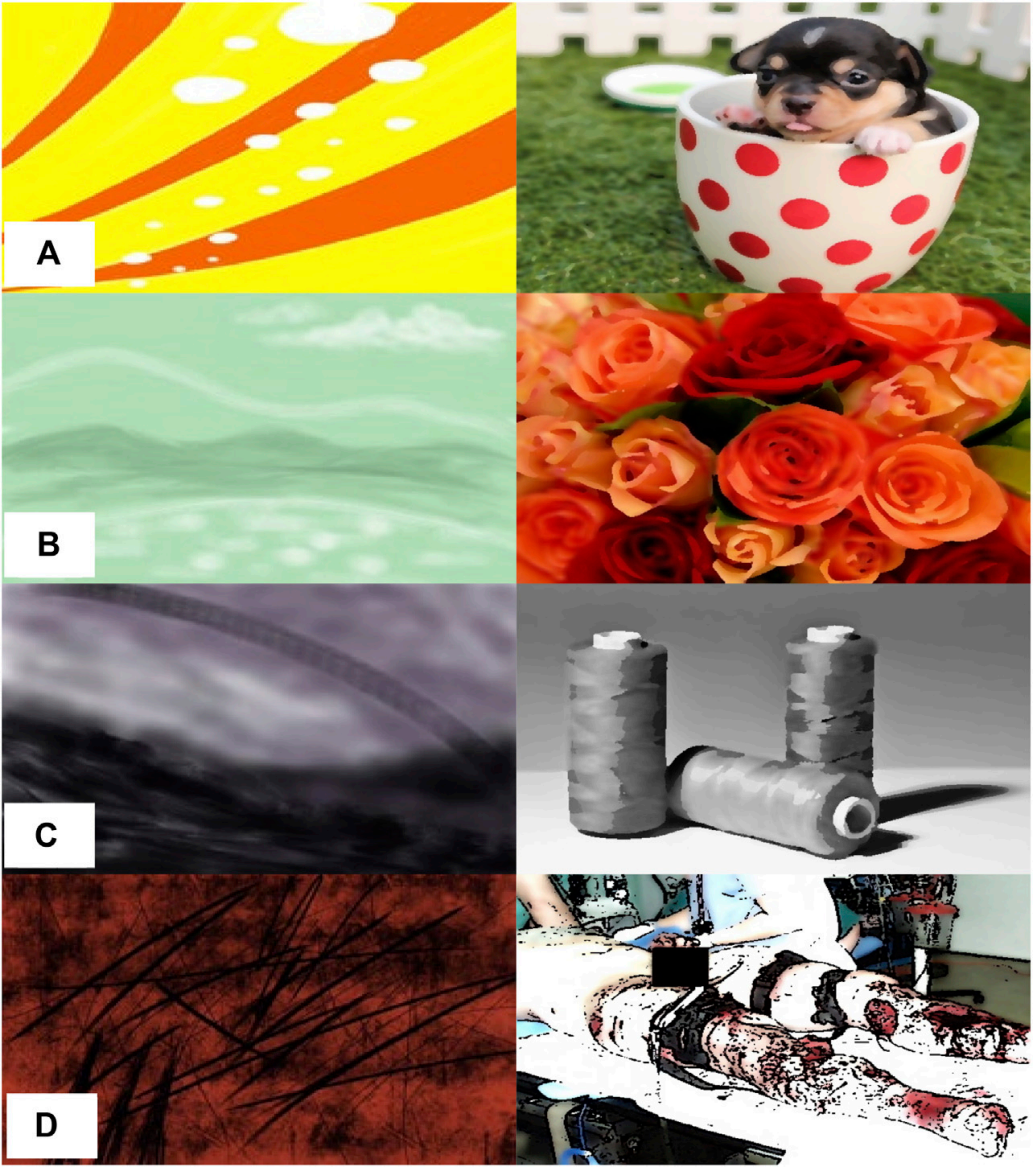
Generic sketches: (left) abstract, (right) representational; **(A–B)** happy, **(C–D)** relaxed, **(E–F)** sad, **(G–H)** angry.

**FIGURE 6:**
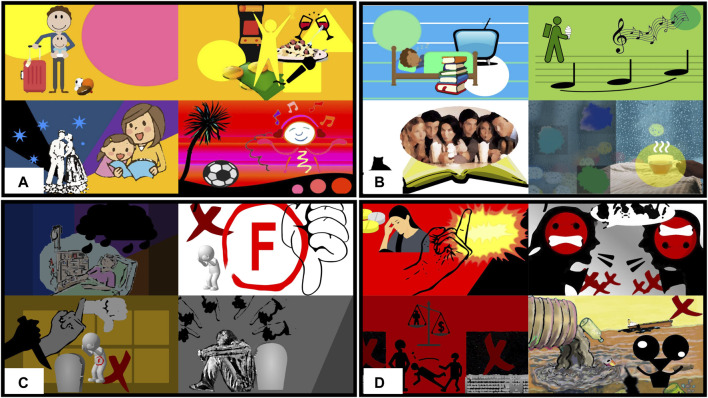
Personalized sketches for four participants: **(A)** happy, **(B)** relaxed, **(C)** sad, **(D)** angry.

After generating the sketches, the participants were invited to complete the follow-up survey. This involved assessing 16 images (personalization (yes or no) vs. art type (abstract or representational) vs. four emotions). First, participants were asked to describe their current emotional state and if they had knowledge about art, to identify potential outliers; i.e., participants who were in an uncommon emotional state or who indicated high artistic knowledge that could involve strong preconceptions. Then the participants conducted four comparisons, once per emotion, in which they ranked the four images for each emotion (personalized/representational, personalized/abstract, generic/representational, generic/abstract) in order of how much the images expressed each emotion (happy, relaxed, sad, angry). Additionally, as a check to see how the participants perceived individual sketches, the Self-Assessment Manikin (SAM) was used to rate the valence and arousal of each sketch before comparisons (Bradley and Lang, 1994); the SAM is a tool for categorizing emotional responses to stimuli, which uses some cartoon pictures of a human smiling or frowning, and the presence or lack of some wiggly lines, to illustrate a range of valence and arousal, with the aim to be easily understood by laypersons. Image orders were randomized, and each participant received a personalized form for the follow-up survey. Figure 5 shows the eight generic sketches used, and Figure 6 shows 32 personalized sketches also generated based on the self-disclosed data from participants.

The first step in analysis was to identify potential outliers based on the preliminary questions about emotional state and artistic knowledge. One outlier was detected: in contrast to three participants who felt well, the remaining participant stated that they felt “depressed.” Furthermore, although none of the participants indicated strong agreement that they have artistic knowledge, the depressed participant’s self-rating was the highest in the group (6, compared to the average of 4.2, SD = 2.2). Examination of the data from the depressed participant indicated some anomalous responses that appeared to be different from those of the other participants and general expectations from the OASIS dataset analysis, and possibly inconsistent and random: For example, the sketch of a small puppy selected by the participant as the happiest of the four images in the comparisons was rated as highly negative via SAM. The generic sketches of an injured bleeding person, as well as the sad and angry generic abstract sketches, were assessed with the most positive score possible. Additionally, a pattern could not be seen in the comparisons, with a different category considered to best express emotion each time. Thus, although there might have been some effect related to artistic preferences, or some external factor such as time pressure, the inconsistencies might have been unintentional due to feeling depressed, which can involve anhedonia and negative fixation (in this case, possibly a loss of joy in seeing positive images, loss of interest in filling out the survey, and interpretation of typically negative symbols such as death by injury as positive).

Based on this, the data were analyzed in two steps, both with and without the outlier data (4 vs. 3 participants). Using all of the data, the personalized representational art was most frequently described as best conveying the intended emotions (75%, 12/16); and, a Chi-squared test with Yates correction confirmed that the participants seemed to perceive the systems differently ($\chi^{2}$(3, N = 16) = 17.8, p = 0.0005), although caution is advised in interpreting this result due to the small number of participants. By contrast, the generic representational art was most frequently described as least conveying the intended emotions (44%, 7/16), and abstract art, generic and personalized, was rated as being in the middle. Also, the most extreme valence and arousal ratings were associated with the personalized sketches. With the consistent data from the 3 non-depressed participants, there was only one case in which the personalized representational art was not indicated as best conveyed the intended emotions (vs. 92%, 11/12), which might have occurred due to ambiguity: this participant disclosed that warm colors seemed both happy and angry, and had indicated abstract symbols such as “ignorance” that might have been difficult to accurately express visually in a sketch. As with all of the data, the most extreme valence and arousal ratings were also associated with the personalized sketches: the most positive valence and arousal scores were given for the personalized representational sketches expressing happiness (1.3 and 2.3 on the 9-point scale), lowest valence for the personalized representational sketch expressing sadness (8.7), and lowest arousal for the personalized abstract sketch expressing relaxation (7.5). Along the way, the variance in scores for representational and abstract art was also checked; for valence, there was more average variance for abstract art than representational art (1.5 vs 0.85), but the case was reversed for arousal (1.5 vs. 2.2).

### 3.4 Bringing Things Together

As noted, the aim of the current article was to start to explore this complex landscape and stimulate discussion, rather than to develop a fully functioning prototype, but nonetheless, a rough proof-of-concept was additionally built to draw together and exemplify some of the insights and concepts developed in the current article: This robot prototype uses general norms, in the form of the visual metaphor concept, to appear contingent and creative, and focuses on symbols that were identified as typical (related to nature, which was part of the online survey, and also rated as one of the most common symbols that can be happy or sad). Furthermore, the robot uses a person’s self-disclosure with representational symbols to personalize its art, which seemed to be easier to use and clearer than abstract symbols for the current context.

Specifically, before the interaction, a person answers some questions about which symbols elicit their emotions. Then the robot starts the interaction with a quick introduction, asks the person to draw a grass field, and records an image of the person’s painting. This analysis results in a judgement if the person’s painting is happy or sad, based on features such as intensity, color, and shapes, as described previously. Then, the robot seeks to express contingency and creativity by painting a different natural scene (e.g., either mountains or sea, depending on the person’s emotional art profile) with a similar emotional feel. Although highly simplified, this interaction brings together some of the concepts discussed in the current article on emotional contingency, creativity, general norms, and personalization, which is also illustrated in a short video demonstration (Cooney, 2020a).

## 4 Discussion

In summary, the current speculative article used a collaborative prototyping approach, including a small user study, to propose a design for a robot to paint with a person in a contingent, creative, and desirable manner, based on personalized visual metaphors. General strategies and personalization are discussed below, followed by identifying some limitations and challenges for future work.

### General Strategies

Some patterns could be identified overall and for representational and abstract art:

• Overall: A central finding in this article was that participants preferred to make art with a balanced system that is both contingent and creative.
• Representational: Also, a small number of 20 frequent categories was identified for representational symbols; the categories most associated with emotions seemed reasonable, because positive effects of physical activity, affection, diet, music, nature, and hobbies are well-known, as well as negative effects of failure, abuse, and injustice. (Appraisal theory also indicates the role of perceived agency in shaping sad vs. angry emotions: abuse can be either impossible or possible to prevent, thereby causing either sadness or anger, whereas reported failures and injustice referred to the respondents themselves, and to others, respectively–here, actor-observer bias suggests that we tend to attribute our own actions more to circumstances outside of our control, and others actions more to potentially avoidable character flaws.)
• Abstract: Some general patterns were also identified for abstract symbols. For colors, the idea that white, black, and gray could express various emotions was not surprising, as black and white art, comprising various shades of gray, is common in media such as comic books, which have been used to express a full gamut of emotions. The association of brown with sadness, although unexpected, also made sense, as brown is a composite color comprising yellow and red with black, which can be thought of as dark orange, and darkness is associated with negative emotions like sadness. However, responses about the emotional meanings of colors did not always agree with Ståhl’s color model; for example, colors in the negative quadrants like purple were considered also to be happy. This could indicate a cultural influence, or a more complex association of a range of various saturations, intensities, and hues with such labels–regardless, it also suggests that such generic guidelines should be taken with a grain of salt, and that personalization can be useful.

For shapes, the finding that circles seem to be positive and triangles to be negative is supported by previous experiments (Heider and Simmel, 1944). Likewise, the observation that horizontal lines can be seen as relaxed and diagonal lines as dynamic (not relaxed) has also been previously indicated (Rodin, 2015). Furthermore, it seems reasonable that diagonal lines would be reported frequently as eliciting emotions because high arousal symbols are more commonly reported than low arousal symbols when seeking to express high or low valence. Also, the lack of consensus regarding vertical lines and squares suggested that additional emotional categories might be required to gain insight into their emotional meanings.

### Personalization

A Folksonomic-style model was used to gather 20.6 self-disclosed labels per person (433 labels for 21 people) for personalization, avoiding the need to know ahead of time what categories to use; participants' self-disclosed perceptions of emotions in art were observed to be highly idiosyncratic and varied greatly–in line with previous work.

• Overall: A central finding was that personalized representational symbols seemed easier to use and clearer than abstract symbols.
• Easier to use: Participants disclosed more information about representational than abstract symbols (2.5 labels, vs. 1.3 labels per question), possibly because fewer abstract categories exist. Furthermore, although all participants were able to describe representational symbols, some participants were not able to describe colors or shapes that expressed an emotion.
• Clearer: Representational symbols also seemed to be more monosemic, and have less overlap between positive and negative emotions than abstract symbols. It seems encouraging that 10 typical categories, and 28 one-label categories, were uniquely tied to one emotion, suggesting that such emotional communications will be perceived as intended. As well, there is some ambiguity associated with abstract symbols; for example, a circle could remind someone of either positive symbols like an angel’s halo, candy, or a soft pillow, or negative symbols like a pit, an open garbage can, or a shark’s mouth gaping wide to violently rip into its prey. Intuitively, this seems to be supported by how humans interact in everyday conversations: when someone asks how we are, we usually mention specific experiences, like doing well on an examination or feeling bad due to a cold; we don’t usually talk about feeling good or bad due to a color or form. Additionally, the small follow-up survey appeared to indicate that for some participants, self-disclosure can be used to better communicate emotions through personalized representational art, rather than personalized abstract art or generalized representational art.

### Limitations and Future Work

Next steps will include conducting more rigorous user studies, overcoming practical challenges, dealing better with some people who for whom personalization seems to be more difficult, improving personalization of abstract art, and exploring other applications:

• Explorative results. These results are limited by the speculative, exploratory approach. As noted, the current article was not intended to provide some final answer as to how robots can interact with people in an emotional and creative way; rather, the aim was to explore some early stage ideas for such a design and stimulate discussion that could aid the design of a variety of future systems, as in some previous articles that have followed a speculative design approach (DiSalvo et al., 2003; Luria et al., 2020). In the experiment conducted, a wide range of cultures and nationalities was represented, the sample range in age was large, and the numbers of female and male participants were unequal. However, it is known that culture, age, and gender and various other factors can affect visual preferences, as in the examples shown in Table 4. Now that some basic insight has been obtained, it would be beneficial to conduct user studies with larger, more uniform groups of participants, also not just from the field of engineering, to elucidate the effects of such factors. Moreover, an autonomous robot system can be used instead of a Wizard of Oz approach. This could be in a lab, or better still, in the “wild.”
• Practical and technical challenges. Prototyping indicated that a current bottleneck is timely generation of novel images that does not require a human in the loop to identify appropriate images to paint; smaller problems include noise in real human environments related to lighting and motion, as well as a seeming lack of easily reusable code for painting robots to render images, possibly due to the high diversity of robot morphologies.
• Abstract art. Another challenge is that it seemed more difficult to personalize abstract art than representational art. Three potential causes suggested themselves: 1) Personalized preferences for abstract art often overlapped with general guidelines–e.g., red and black colors with diagonal lines to represent anger–whereas, the space for representational art is much more expansive; for example, no participant described the contents of the generic image, a small puppy in a cup in a grass field, when queried for a happy symbol. 2) Another potential cause is that the participants might not have known ahead of time what kind of abstract art would make them feel a certain way, which was indicated in some comments. 3) Finally, our measurements in the sparse, open-ended survey might not have been sufficient to model participants' preferences. For 2), a reflective approach to personalization, intended to empower users by querying to encourage thought about goals before starting an activity, might be useful (Lee et al., 2015). (Another interesting observation in the same work was that, although robot designers typically try to avoid boring people with repetition, human experts suggested the importance of repeatedly querying users to uncover hidden motivations.) Additionally, Big Five analysis (John et al., 2008) could be used to stereotypically infer a person’s perception of art: e.g., if positive emotions could be experienced by linking conscientiousness to clean lines and shapes, openness to more novel art, or extraversion to stronger colors and color contrasts. For 3), aside from introducing more questions and considering other factors such as composition, interactive personalization could be used throughout a more extended period (Clabaugh, 2017), possibly like the series of questions in an eye exam; similar to the above work by Lee et al., despite foreseeing a possibility of survey fatigue, participants in this work also reported enjoying being prompted frequently.
• Difficult individuals. Some participants seem to be easier to make art for than others; specifically, participants who listed more representational symbols, colors, and shapes that elicit emotions, while not using the same symbols for multiple emotions, and providing sufficient information to be able to visually depict images: Typically, paintings use more than one color or shape, so it might be easier to prepare art for participants who listed more options. Likewise, it might be easier to express emotions in artwork for participants who did not say that one color or shape expressed multiple emotional meanings to them. Additionally, some participants referred to personal information, like “my family,” which alone could be insufficient for visual depictions. Another challenge was noted with the seemingly inconsistent appraisals by the depressed participant; in a therapeutic context, robots will probably frequently interact with people with depression; therefore, such persons should not be excluded or marginalized, but rather centralized in at least some human models.

**TABLE 4:** Examples of effects of culture, age, gender on visual aesthetics.

	Representational	Color	Shape	References
Culture	Swastikas can be a positive symbol for buddhism in the east or a negative symbol of the horrors of war in the west	Red is associated with communism, which could be interpreted positively or negatively	Aesthetic preferences for simplified, imperfect lines in Japanese wabi-sabi have been contrasted with a western preference for perfect, controlled shapes	Holman and Vertegaal, (2008)
Age	Elderly can prefer skeuomorphic rather than flat designs; young children might not recognize obsolete symbols such as video rentals, card catalogs, hole-punched floppy disks, and rotary-dial telephones	Elderly typically prefer colors of shorter wavelengths (blue, green, and violet, vs. red, orange, and yellow)	Infants have a visual preference for curved shapes (especially faces similar to their carer, but also shapes like bull’s eyes), and females of reproductive age prefer masculine (square) faces more than females in puberty and post-menopause	Fantz and Miranda. (1975); Holman and Vertegaal. (2008); Little et al. (2010); Birren. (2016)
Gender	Girls typically draw more realistic, docile scenes with nature and fewer objects	Girls typically use more colors than boys, including more blending and harmonious combinations	Girls typically use more curved and fewer rectilinear shapes	Tuman, (1999)

To acquire better data, motivation can be clarified for those participants who responded with only few or overlapping labels: Was there an underlying difference in how they perceive emotions in art or some other confounding reason (e.g., was there an assumption that responses had to be non-overlapping (demand characteristics), or were some tired of the survey and trying to get it done fast)? Survey instructions could then be refined, gaps could be caught at the time of profile creation, or a robot system could query afterwards for more information, although in any case there is a need to be careful about ethics in treating personal information. To determine if a painting will be able to correctly convey an intended emotion to someone who is depressed, one way to seek to avoid miscommunications might be to use a multimodal strategy to better detect and convey emotions: in addition to analyzing art, a robot can check a person’s emotions via a Brain-Machine Interface, and verbally ask for confirmation that these emotions have been correctly identified, before describing its intentions as it paints.

• Other applications. The concepts here could be applicable to other kinds of art, from sculpture to photography, drawing, and other crafts. Moreover, the usefulness of ensuring a balance between exogeny and endogeny, and thereby emotional contingency and creativity, might not be restricted only to painting robots; rather a similar pattern might be useful for interacting with humans in various contexts, such as advertisement, writing, music, and games. For example, the author of the current article was part of a team that set up an android in a department store as a kind of lifelike, moving mannequin in a Valentine’s Day display for two weeks in February 2012 (Geminoid F, at Takashimaya in Shinjuku, Tokyo) (Mar 2017); the android’s code sought to balance reacting to people who came close and waved, with having her own agenda, like looking at her smartphone or absentmindedly to the side, with varying emotions. In writing haikus, there is often a “timely” exogenous component shaped by a poet’s perception of a moment, as well as a “timeless” endogenous component, revealing the inner life of the object of the poem (Higginson and Harter, 1989). In music, improvisations can involve reactive exogenous skills, e.g., to stay aligned with a change of rhythm, and endogenous compositional skills, e.g., to flesh out a musical fragment (Alperson, 2010). In playing games with a human, positive effects have also been observed for robots that combine exogenously reacting in a large, meaningful manner, with exhibiting its own consistent endogenous intentions (Cooney and Sant’Anna, 2017). Future work will involve identifying other contexts where such a design could be useful.

Consideration of such topics could allow such robots to exert a positive influence on interacting humans.

## 5 Conclusion

This article suggested the “smart phone hypothesis,” that social robots will become accepted into various human environments when they become capable of interacting in a variety of useful ways, including within challenging applications involving emotions and creativity, like art-making. A speculative approach involving collaborative prototyping with artists and engineers, along with a small user study, provided some insight into practical challenges such as timely autonomous image generation, as well as general strategies and personalization:

• General: participants would prefer to make art with a robot that is both emotionally contingent and creative, rather than merely one or the other, which can be done by balancing exogeny and endogeny; also, some shared patterns could be identified for both representational and abstract symbols, such as that personalizable symbols such as sports, food, family, and nature are perceived in a positive way
• Personalized: participants' self-disclosed perceptions of emotions in art were highly idiosyncratic and varied greatly, in line with observations in previous work, suggesting also that some participants' perceptions might be easier to model than others; also, representational symbols appeared to be easier to use for personalization than abstract symbols, in terms of encouraging more disclosure, being less ambiguous and more easily related to individual emotions, and seeming to more clearly convey emotions in some sketches.

These results were discussed with the aim of stimulating ideation, which included proposing some next steps in terms of reliability, practicality, challenging cases, art forms, and other applications.

The basic contribution is insight into some considerations for art robots that could help to support well-being in interacting people. At a higher level, exploration in this research direction could potentially facilitate technological acceptance for robots in human spaces, and also eventually provide an opportunity for us to learn about emotions and creativity, two phenomena which are tightly intertwined in our natures as humans.

## Figures and Tables

**FIGURE 7:**
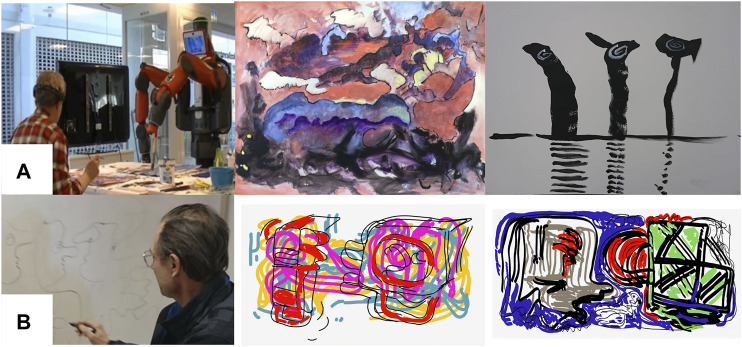
Two professional artists assisted in our exploration by providing advice and creating examples of emotional art, e.g.: **(A)** some art by Dan Koon showing use of color and form, **(B)** some art by Peter Wahlbeck showing positive and negative emotions.

## Data Availability

The datasets presented in this article are not readily available because emotional perceptions could potentially be used to identify individuals. Partial data for which this is not a concern could be made available. Requests to access the datasets should be directed to martin.daniel.cooney@gmail.com.

## Appendix A: Our Team

Since starting in 2017, 63 people have been directly involved: two artists, two researchers, one PhD student, 26 master’s students (two in particular), two undergraduate students, and 30 experiment participants. More people have been involved indirectly, comprising at least 30 observers to a free demo event; classes of students and researchers who discussed thesis results; and people who have read, listened to, or watched various newspaper articles, radio appearances, and thirteen YouTube videos showing this work with robot art, which have also been viewed over a thousand times as of October 2020.

The two artists on the team were Dan Koon and Peter Wahlbeck: Dan Koon is an American artist and author living in southern Sweden, who was self-trained from copying masters such as Rembrandt, Vermeer and Monet. He uses mainly acrylics and iPad sketches to seek to portray nonmaterialistic aspects of the individual, such as existence and creation. Peter Wahlbeck is a Swedish artist, comedian, and actor, who has been making art for over 30 years. He likes to make people happy and get them to laugh, which can be seen in the vibrant, colorful and creative characters that appear throughout his work. His paintings, although intended more to decorate than to convey political messages, yet seek to stimulate thought. Some of their art is shown in Figure 7.
